# Nitric oxide synthase phosphorylation in fetoplacental endothelium is enhanced by agonism of Piezo1 mechanosensor in small for gestational age babies

**DOI:** 10.1530/RAF-22-0100

**Published:** 2023-01-18

**Authors:** L C Morley, M Debant, H J Gaunt, N A B Simpson, D J Beech

**Affiliations:** 1 Leeds Institute of Cardiovascular and Metabolic Medicine, LIGHT laboratories, University of Leeds, UK; 2 Academic Department of Obstetrics and Gynaecology, Level 9 Worsley Building, School of Medicine, University of Leeds, Leeds, UK

**Keywords:** placenta, endothelial cells, Piezo1, shear stress, nitric oxide

## Abstract

Friction caused by blood flowing across cells that line blood vessels (endothelial cells) activates sensors of mechanical force. This produces nitric oxide (NO) which widens placental blood vessels, enabling more blood flow to the baby. This study sought to determine whether the mechanical sensor, Piezo1, is important for NO production in fetoplacental endothelial cells (FpECs) and whether the steps in this pathway are different in small for gestational age (SGA) babies, where placental blood flow is often altered. We showed that in healthy FpECs, blood flow increased NO signalling. We suggest that in SGA babies, FpECs have an increase in baseline levels of NO signalling, suggestive of a compensatory drive. Treating healthy and SGA cells with a Piezo1 chemical activator, Yoda1, upregulated NO signalling. This shows that Piezo1 is linked to NO and that in SGA, FpECs have the capacity to further increase NO. Further research will establish whether Piezo1 enhancement leads to increased blood flow in the placenta. If so, Piezo1 could be a new target for developing treatments to prevent poor growth of babies in the womb.

## Research

Babies are born small for gestational age (SGA) in up to 10% of pregnancies, which can have both immediate and long-term clinical consequences (Audette & Kingdom 2018). The condition is multifactorial but is commonly associated with altered blood flow within the placental circulation.

Fetoplacental endothelial cells (FpECs) are constantly exposed to fluid shear stress (FSS) by blood flow. This FSS is the most powerful stimulus for the production of nitric oxide (NO) via endothelial NO synthase (eNOS), which is well-known to induce placental vasodilatation (Learmont & Poston, 1996, Sprague *et al.* 2010). Detailed knowledge of the mechanism by which FSS leads to NO synthesis in humans is lacking.

Our group investigated the FSS sensor, Piezo1. This Ca^2+^-permeable ion channel is increasingly recognised as important for vascular adaptation in multiple body systems. We demonstrated a reduction in FSS-evoked eNOS in commercially sourced pooled human umbilical vein endothelial cells (HUVECs) after *PIEZO1* silencing, suggesting that Piezo1 had a regulatory role through NO (Li *et al.* 2014). We showed that* PIEZO1* was consistently expressed in FpECs cultured from human placentas, and activation with the small molecule channel agonist, Yoda1, increased intracellular Ca^2+^ (Morley *et al.* 2018).

We sought to determine whether Piezo1 activity led to NO signalling and if there were differences between healthy placentas and those where the baby had been SGA. FpECs were cultured from the placentas of patients undergoing elective caesarean sections at Leeds Teaching Hospitals NHS Trust, as previously described (Morley *et al.* 2018). Patients were recruited as either SGA or appropriately grown for gestational age (AGA). SGA was defined as birthweight < 10th percentile according to UK World Health Organisation Growth Charts.

**Figure 1:**
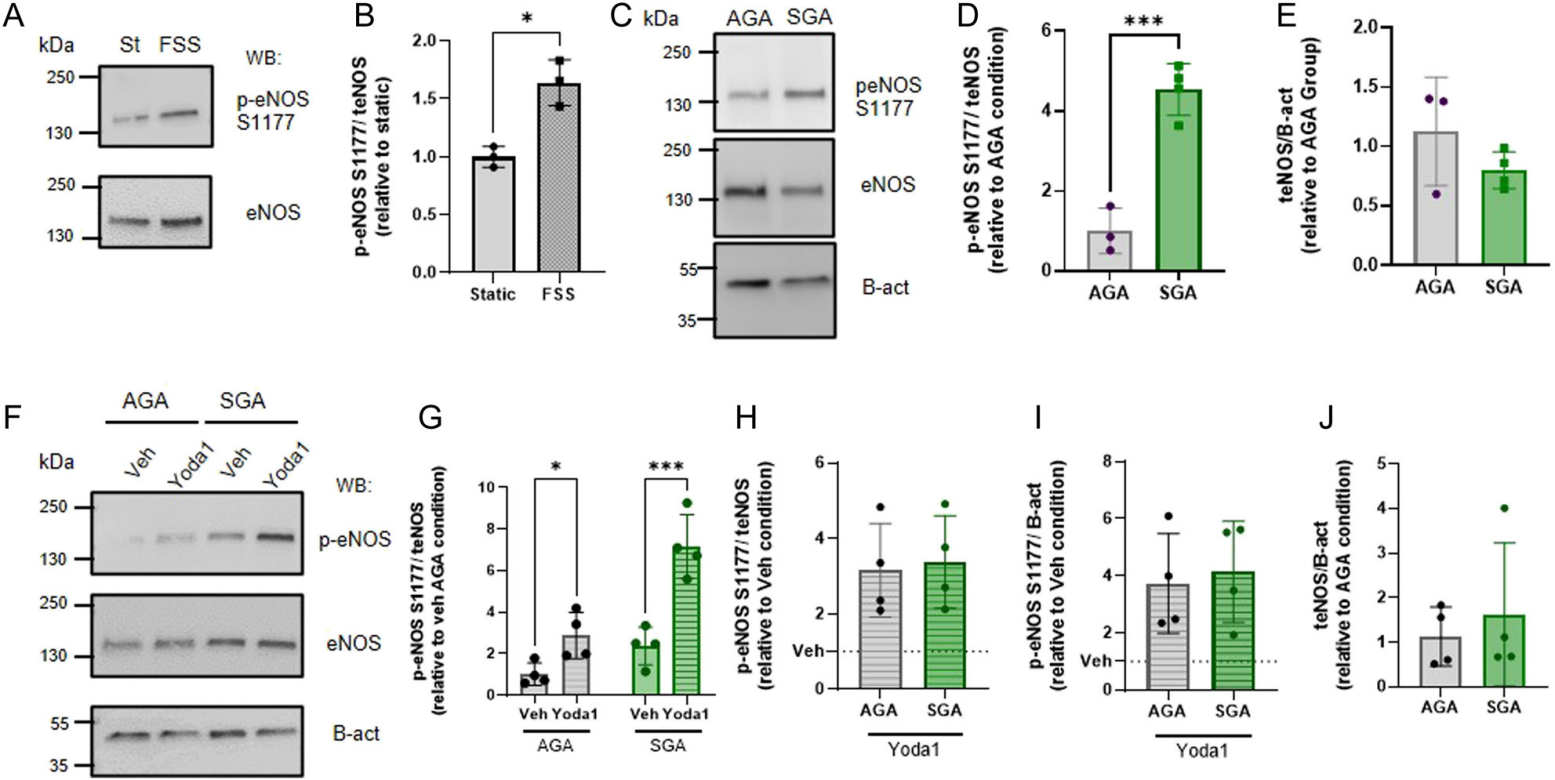
SGA FpECs show increased basal eNOS S1177 phosphorylation, and Yoda1 increases p-eNOS in AGA and SGA FpECs. (A) Representative Western blot (Wb) labelled with anti-phospho(p)-eNOS (S1177) and anti-eNOS antibodies for AGA FpECs exposed to 10 dyn/cm^2^ FSS on an orbital shaker (FSS) or not (Static, St). (B) Quantification of data of the type exemplified in (A), showing mean ± s.d. for p-eNOS intensity relative to total eNOS (teNOS), normalised to static condition (*n* = 3). (C) Representative Wb labelled with anti-p-eNOS (S1177), anti-eNOS antibodies and anti-β-actin for FpECs isolated from AGA or SGA samples (*n* = 3/4, *N* = 2 replicates, respectively). (D-E) Quantification of data of the type exemplified in (C), showing mean ± s.d. for p-eNOS intensity relative to teNOS (D) or teNOS intensity relative to β-actin (E) normalised to AGA. (F) Representative Wb labelled with anti-p-eNOS (S1177), anti-eNOS and anti-β-actin for FpECs isolated from AGA or SGA samples and exposed to 2 µM Yoda1 for 1 min or vehicle (Veh) control (DMSO). (G-H) Quantification of data of the type exemplified in (F), showing mean ± s.d. for p-eNOS intensity relative to teNOS normalised to AGA Veh condition (G) or to respective Veh condition (H). (I) teNOS intensity relative to β-actin normalised to AGA Veh (*n* = 4 each group). Superimposed dots are the individual underlying data values for each individual experiment. ^*^P < 0.05, ^***^P < 0.001 vs stated condition. Statistical significance was evaluated using two-way RM matched ANOVA followed by Šidάk’s *post hoc* test for multiple comparisons using Welch’s *t-*test.

FpECs from AGA samples exposed to FSS were probed with antibody to phosphorylation (p) at serine site 11777 (S1177) in endothelial NO synthase (eNOS), which is linked to eNOS activation. When compared to the static control, FpECs exposed to FSS had greater p-eNOS relative to total eNOS (teNOS, *P* = 0.018, Fig. 1A and B).

Basal levels of p-eNOS were compared between AGA and SGA samples. This demonstrated a significant increase in p-eNOS in SGA lysates when normalised to teNOS (*P* = 0.0007, Fig. 1C and D). There was no evidence of a difference in teNOS in the SGA samples vs AGA, when normalised to β-actin (relative to AGA, *P* = 0.34, Fig. 1E).

Yoda1 was applied to FpECs, and lysates were probed with anti-S1177 p-eNOS and anti-eNOS antibodies as described above. In AGA FpECs, Yoda1 increased p-eNOS vs vehicle control (DMSO) relative to teNOS (*P* = 0.031, Fig. 1F and G). In SGA FpECs, Yoda1 treatment also significantly increased p-eNOS compared to vehicle control (normalised to teNOS, *P* = 0.0003, and Fig. 1F and G). The intensity of p-eNOS induced by Yoda1 application did not significantly differ between the AGA and SGA groups when normalized to either teNOS or β-actin (*P* = 0.811 and *P* = 0.752, Fig. 1H and I, respectively). In addition, there was no evidence of a difference in teNOS between the groups after Yoda1 treatment (*P* = 0.603, Fig. 1J).

These findings demonstrate coupling between Piezo1 and p-eNOS in FpECs. We show that Yoda1 phosphorylates the S1177 regulatory site on eNOS in the fetoplacental endothelial cells in both AGA and SGA placentas, mimicking flow-induced p-eNOS. We propose that basal p-eNOS undergoes upregulation in SGA. The basal NO signalling pathway is not saturated in these cells, however, Yoda1 led to further enhancement of p-eNOS. This raises the question of whether Piezo1 agonism could be a novel intervention for SGA treatment, by promoting the production of the key vasodilator, NO.

## Declaration of interest

The authors declare that there is no conflict of interest that could be perceived as prejudicing the impartiality of the research letter.

## Funding

LCM was funded by an MRC and RCOG funded Clinical Research Training Fellowship. The research was also supported by a British Heart Foundation Programme Grant to DJB (RG/17/11/33042), a Wellcome Investigator Award to DJB (110044/Z/15/Z) and a British Heart Foundation PhD Studentship to HJG (FS/14/22/30734). For the purpose of Open Access, the authors have applied a CC BY public copyright licence to any Author Accepted Manuscript version arising from this submission.

## Ethical approval

Patients delivering by elective caesarean section at Leeds Teaching Hospitals Trust were provided with written information and consent was obtained, in accordance with the approval granted by the local ethics committee (Ref 18/LO/0067).

## Author contribution statement

LCM, NABS and DJB designed the study and generated research funds. LCM, HG and MD performed the experiments and analysed the data. LCM and MD wrote the paper. All authors edited and approved the manuscript.
